# Tracheal Diverticulum: A Unique Case with Intraoperative Morphologic Assessment

**DOI:** 10.1155/2015/167394

**Published:** 2015-07-28

**Authors:** Rohini Chennuri, Pavithra Dissanayake, Urjeet A. Patel, Gabor Tarjan

**Affiliations:** ^1^Department of Pathology, Cook County Hospital, Stroger, 1901 W. Polk Street No. 603, Chicago, IL 60612, USA; ^2^Division of Otolaryngology-Head & Neck Surgery, Department of Surgery, Cook County Hospital, Stroger, 1901 W. Polk Street No. 603, Chicago, IL 60612, USA

## Abstract

There are rare case reports of tracheal diverticula or paratracheal air cysts. These cases, however, were reported mostly as incidental sonographic or radiologic findings without histologic confirmation. Furthermore, the handful of studies that describe this entity histopathologically report only cases in patients with prior respiratory symptoms. Here, we report a rare case of an asymptomatic 60-year-old female with no significant past medical history who presented with primary hyperparathyroidism. She was found to have an incidental right paraesophageal air-filled diverticulum with multiple thin septations on her imaging studies. She was taken to surgery and the histologic examination of the specimen revealed multiloculated cystic cavity lined by respiratory-type columnar epithelium with lymphocytic infiltrate and minor salivary glands within the surrounding stroma, rendering the diagnosis of tracheal diverticula.

## 1. Introduction

There are rare case reports of paratracheal air cysts/tracheal diverticula. These are generally incidental sonographic or radiologic findings without subsequent morphologic diagnosis. Even the classic histopathologic description is based only on autopsy studies. Hereby, we report a unique case in which the histopathologic diagnosis of a tracheal diverticulum was considered intraoperatively and confirmed on the final surgical specimen in a 60-year-old woman who underwent surgery for hyperparathyroidism.

## 2. Case Report

**Figure 1:**
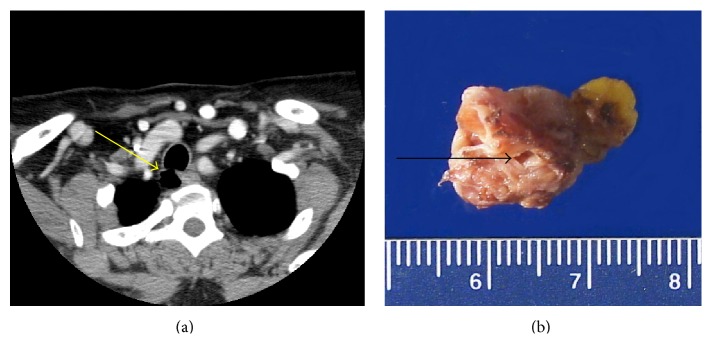
CT scan of the neck w/contrast showed a right paraesophageal air-filled diverticulum extending from the trachea (a). Grossly the specimen consisted of a 2.4 × 1.5 × 0.8 cm tan brown cystic structure with an opening of 0.2 cm in diameter (marked by arrow) and attached adipose tissue (b).

A 60-year-old female with no significant past medical history presented with primary hyperparathyroidism. She was asymptomatic with slightly elevated serum calcium and parathyroid hormone (PTH) levels. The sestamibi scan did not show any uptake, while the computed tomographic scan (CT) of the neck showed an incidental right paraesophageal air-filled diverticulum with multiple thin septations (Figure 1(a)). During surgery, three and a half parathyroid glands were explored and resected resulting in the drop of blood PTH level, confirming the diagnosis of parathyroid hyperplasia. In addition, a cystic structure densely adherent to the trachea was found in the right tracheoesophageal groove. Blunt dissection was performed around this mass without difficulty, though tissue planes between the mass and the trachea could not be readily separated. Sharp dissection was then performed to complete the resection off the tracheal wall over the small surface area to which it seemed attached. There did not appear to be any apparent lesion left attached to the trachea and there was no tracheal injury or entry point noted at this time. The specimen was sent for intraoperative assessment, which revealed several lumina/cystic spaces lined by ciliated columnar epithelium with mild lymphocytic infiltrate and salivary-type glands in the adjacent fibrous tissue. Given the absence of apparent tracheal entry point, no formal repair of the trachea was undertaken, though the patient was monitored postoperatively for any signs of tracheal communication. In the absence of detailed clinical information, based on these histologic findings, a tracheal diverticulum, laryngocele, or bronchogenic cyst was favored. Lymphoepithelial cyst and branchial cleft cyst were also considered in the differential diagnosis.

The specimen submitted for pathologic examination consisted of a 2.4 × 1.5 × 0.8 cm tan brown multiloculated/cystic structure with some attached adipose tissue. The cyst wall measured 0.1 cm in thickness. An opening of 0.2 cm in diameter was noted on one aspect of the lesion (Figure 1(b)).

**Figure 2:**
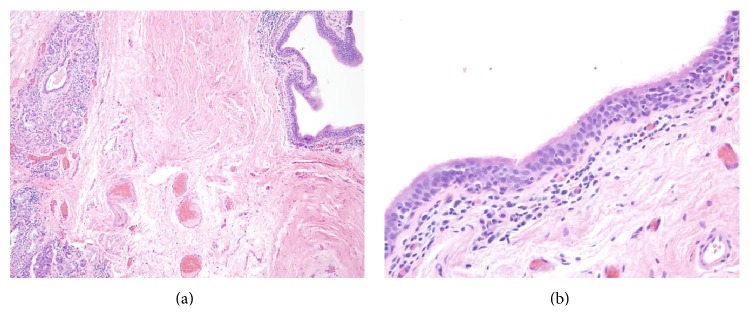
Lesion composed of cystic spaces surrounded by inflammatory infiltrate and adjacent minor salivary gland tissue ((a): HE ×100). Cystic spaces were lined by respiratory-type ciliated columnar epithelium with diffuse mild lymphocytic infiltrate ((b): HE ×400).

Histologic examination of the entire lesion confirmed the presence of cystic spaces lined by respiratory-type ciliated columnar epithelium, minor salivary gland tissue, and lymphoid aggregates (Figures 2(a) and 2(b)). Given the morphologic, imaging, and operative findings, the diagnosis of tracheal diverticulum was rendered. Branchial cleft cyst and bronchogenic cyst were also considered in the differential diagnosis but were not favored for reasons mentioned in the discussion.

## 3. Discussion

Paratracheal air collections are usually found as incidental findings on imaging studies. In these cases, the differential diagnosis includes tracheal diverticulum, laryngocele, pharyngocele, Zenker's diverticulum, apical hernia of the lung, and apical bullae [1]. Pharyngoceles and Zenker's diverticula can be identified by barium study, apical hernia and apical bullae of the lung can be identified on CT, and laryngocele can be localized as the dilated saccule of the laryngeal ventricle. Paratracheal air cyst can also be misdiagnosed as a parathyroid mass on ultrasonography [2]. These lesions generally do not undergo morphologic examination. The very few histologic reports of surgically confirmed paratracheal air cysts revealed ciliated columnar epithelium [3, 4]. This is in line with the most elaborate autopsy study of MacKinnon [5]. He found 8 cases of tracheal diverticula in 867 routine serial autopsies rendering the prevalence approximately 1%. MacKinnon described ovoid, pedunculated, or sessile multilocular cysts, measuring 0.5 to 3.0 cm in longest diameter, attached to the right posterolateral border of the trachea. The upper border of these cysts was found below the right lobe of the thyroid gland. The age incidence was 30–80 years, with male predominance (7/10), all with chronic respiratory disease. The cysts communicated with the tracheal lumen by one or more openings and had a wall of dense fibrous tissue, usually found adherent to the neighboring structures. Histologically, the cysts were lined by stratified columnar ciliated epithelium, with chronic inflammation and mucous glands in the wall, with the absence of smooth muscle and cartilage. These acquired tracheal diverticula are thought to be produced by mucosal herniation as a result of elevated intraluminal pressure. The almost exclusive location of these lesions on the relatively unsupported right side of the trachea may be related to its shifting to the right from the esophagus. MacKinnon opined that raised intratracheal pressure with a history of chronic respiratory disease and cough with tracheitis led to hyperplasia of the tracheal mucous glands and development of the tracheal cysts [5]. Our patient, in contrast, did not have any condition known to increase the luminal pressure.

Congenital tracheal diverticula are different from the acquired ones and are thought to be malformed supernumerary branches of the trachea [6]. They resemble complete tracheal structures including cartilage and smooth muscle in addition to the respiratory epithelium and mucus glands. Similarly, bronchogenic cysts of the mediastinum also harbor these tissue elements. However, although they tend to arise on the right side of the trachea similar to the tracheal diverticula, they have no communication with the lumen of the trachea.

Branchial cleft cysts are developmental cysts considered synonymous by some with the cervical lymphoepithelial cyst. They may be lined by columnar epithelium (and squamous epithelium) resting on lymphoid tissue. They most commonly present in the neck; however, those arising from the fourth branchial arch may occur in the mediastinum [7]. However, they are not connected to the trachea and salivary-type glands would be an unusual finding.

In summary, we presented a unique case of tracheal diverticulum with intraoperative morphologic assessment. This entity, however rare, needs to be kept in mind when encountering a “paratracheal air cyst” because symptomatic or infected diverticula need surgical resection [3].
